# Optimization of Gelatin and Crosslinker Concentrations in a Gelatin/Alginate-Based Bioink with Potential Applications in a Simplified Skin Model

**DOI:** 10.3390/molecules30030649

**Published:** 2025-02-01

**Authors:** Aida Cavallo, Giorgia Radaelli, Tamer Al Kayal, Angelica Mero, Andrea Mezzetta, Lorenzo Guazzelli, Giorgio Soldani, Paola Losi

**Affiliations:** 1 Institute of Clinical Physiology, National Research Council, 54100 Massa, Italytamer.alkayal@cnr.it (T.A.K.); giorgio.soldani@cnr.it (G.S.); paola.losi@cnr.it (P.L.); 2 Department of Pharmacy, University of Pisa, 56126 Pisa, Italy; angelica.mero@farm.unipi.it (A.M.); andrea.mezzetta@unipi.it (A.M.); lorenzo.guazzelli@unipi.it (L.G.)

**Keywords:** gelatin-based bioink, calcium chloride crosslinker, 3D bioprinting, skin model

## Abstract

Three-dimensional bioprinting allows for the fabrication of structures mimicking tissue architecture. This study aimed to develop a gelatin-based bioink for a bioprinted simplified skin model. The bioink printability and chemical-physical properties were evaluated by varying the concentrations of gelatin (10, 15, and 20%) in a semi-crosslinked alginate-based bioink and calcium chloride (100, 150, and 200 mM) in post-printing crosslinking. For increasing the gelatin concentration, the gelatin-based formulations have a shear thinning behavior with increasing viscosity, and the filament bending angle increases, the spreading ratio value approaches 1, and the shape fidelity and the printing resolution improve. However, the formulation containing 20% of gelatin was not homogeneous, resulting also in poor printability properties. The morphology of the pores, degradation, and swelling depend on gelatin and CaCl_2_ concentrations, but not in a significant way. The samples containing 15% of gelatin and crosslinked with 150 mM CaCl_2_ have been selected for the bioprinting of a bilayer skin model containing human fibroblasts and keratinocytes. The model showed a homogeneous distribution of viable and proliferating cells over up to 14 days of in vitro culture. The gelatin-based bioink allowed for the 3D bioprinting of a simplified skin model, with potential applications in the bioactivity of pro-reparative molecules and drug evaluation.

## 1. Introduction

In recent years, according to the 3Rs principles of replacement, reduction, and refinement and as a consequence of European Union (EU) Directive 2010/63/EU, which banned animal testing for cosmetic applications, the demand for alternative methods to animal model is constantly increasing [1,2,3]. The development of in vitro skin models received great attention from researchers as well as from chemical, pharmaceutical, and cosmetic industries because in vivo animal testing, which is time-consuming and expensive, is often ineffective due to the anatomical differences between animal and human skin [4,5].

In this context, the 3D bioprinting technology, compared to the traditional tissue engineering approaches, offers the possibility of an automated manufacturing process that enables the biofabrication of complex structures controllingcontrol the deposition of a biomaterial, known as bioink, loaded with one or more cell types and/or bioactive molecules [6,7]. After bioprinting, a bioink can retain its structure due to crosslinking via chemical, physical, and enzymatical bonds [8,9].

The bioink formulation is a crucial aspect of the 3D bioprinting process. In particular, the bioink needs to be compatible with the extrusion technology, and its rheological properties, such as shear thinning behavior and viscosity, should guarantee cell survival during the printing process. In addition, the bioink should be biocompatible and mimic the tissue extracellular matrix to support cell viability and proliferation during the in vitro culture period. The bioink properties affect the printing resolution, stability, and integrity of the created structures [3,10,11].

Several biomaterials, especially polymers of natural origin, such as gelatin, collagen, and hyaluronic acid, have been investigated to support cell adhesion in bioink formulation [12]. Gelatin-based bioinks are receiving great attention (31% of formulations investigated until now), and their main application modality (71%) is represented by extrusion bioprinting [13]. Gelatin is a low-cost biodegradable protein obtained via the thermal denaturation or controlled hydrolysis of collagen, and it is a thermosensitive biomaterial that undergoes physical gelation below room temperature [14,15,16]. Gelatin possesses the tripeptide Arg-Gly-Asp (RGD) sequence as important binding moieties for cell adhesion, has low antigenicity, and contains peptide sequences that can be cleaved by the matrix metalloproteinases enzymes facilitating the remodeling of the microenvironment by encapsulated cells. Moreover, gelatin promotes cell migration, differentiation, and proliferation due to viscoelastic properties [17]. However, gelatin has low viscosity and unstable physical gelation, causing poor printability, weak shape fidelity, and low mechanical strength, which limit its application for the fabrication of complex, large-scale, and multicellular constructs [13].

Many strategies have been investigated to improve gelatin printability, such as the methacrylation of the lysine residues [10,11,12,13,14,15,16,17,18,19,20] or the addition of other biomaterials such as alginate [21,22,23], which crosslinks in the presence of calcium ions. In particular, Shi et al. proposed a novel dermal substitute scaffold manufactured with sodium alginate/gelatin composite material [24], while Yao et al. proposed hydrogels based on gelatin and sodium alginate to differentiate epithelial progenitors for sweat gland regeneration [25]. In another study, Kang et al. proposed gelatin/alginate hydrogel to construct a multilayer composite scaffold simulating the HF microenvironment in vivo [26].

The primary aim of the present study was to investigate the effect of incremental gelatin concentration increases in the alginate-based bioink formulation on the rheological properties and printability of bioinks, as well as to assess the effect of different calcium chloride crosslinking solutions on bioink properties such as the morphology, the degradation time, and swelling. Consequently, the formulation which showed superior properties in terms of printability was employed through bioprinting for the fabrication of a simplified skin model containing human primary fibroblasts and keratinocytes, showing viable and proliferated cells up to 14 days.

## 2. Results

### 2.1. Bioink Sterility Assessment

**Figure 1:**
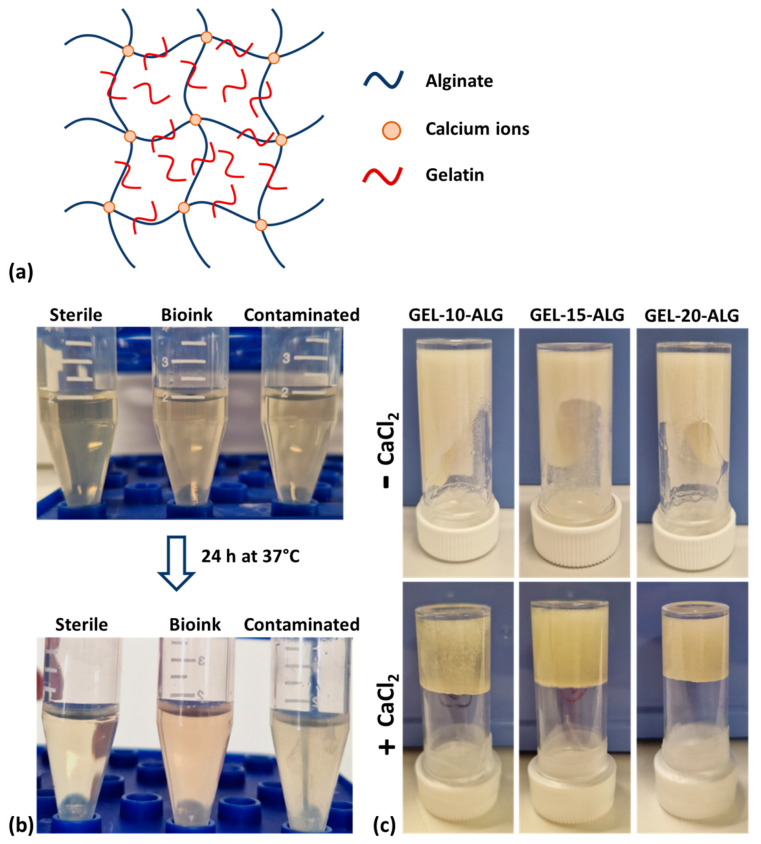
(**a**) Schematic representation of semi-IPN gelatin–alginate bioink; (**b**) bioink sterility assessment; (**c**) vial-inverting method.

The structures of crosslinked bioink obtained by adding small amounts of CaCl_2_ directly into the gelatin–alginate solution and by submersion in a CaCl_2_ solution after bioprinting is a semi-interpenetrating polymer network (semi-IPN), and the structure is represented in Figure 1a.

The bioink sterility was investigated immediately after preparation to validate the protocol employed. The tested formulations were sterile (Figure 1b), and the typical vortex of bacteria observed in the contaminated aliquots was not observed in the bioink aliquot despite an increment of turbidity being observed due to the bioink dissolving into the bacteria culture media. Therefore, the protocol is considered validated.

### 2.2. Rheological Properties and Homogeneity

In Figure 1c, the qualitative effect on the viscosity of the CaCl_2_ addition to the gelatin and alginate solutions is shown. In particular, the alginate and gelatin semi-crosslinked solutions result in more viscous properties than non-semi-crosslinked ones.

**Figure 2:**
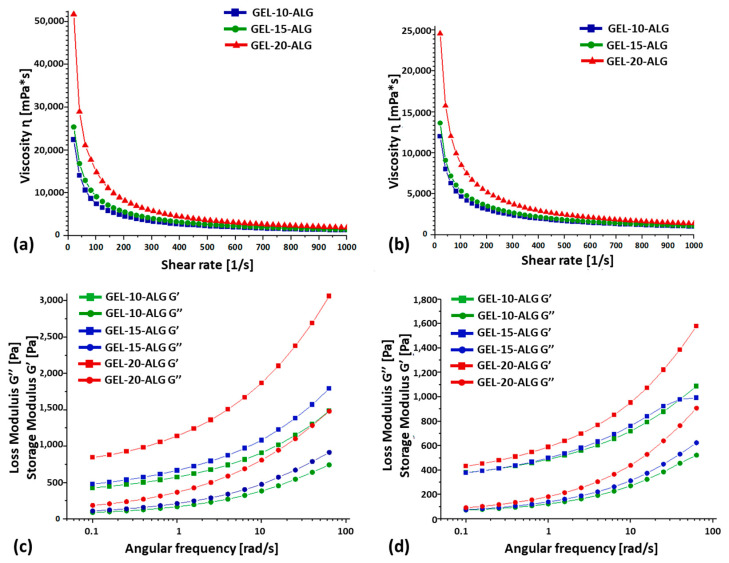
Rheological characterization of GEL-10-ALG, GEL-15-ALG, and GEL-20-ALG at (**a**–**c**) 25 °C and (**b**–**d**) 37 °C. Different scales were reported in (**a**–**d**) graphs.

The results of the rheological characterization of the proposed gelatin-based bioink formulations performed at 25 °C and 37 °C are reported in Figure 2. A decreasing viscosity was measured for the increasing shear rate for all tested formulations. In addition, bioink viscosity increases according to gelatin concentration, while it decreases at 37 °C compared 25 °C. Regarding the storage (G′) and loss (G″) moduli, they are both affected by the gelatin concentration, especially at 25 °C, which represents the room temperature, while similar values have been measured for GEL-10-ALG and GEL-15-ALG at 37 °C.

**Figure 3:**
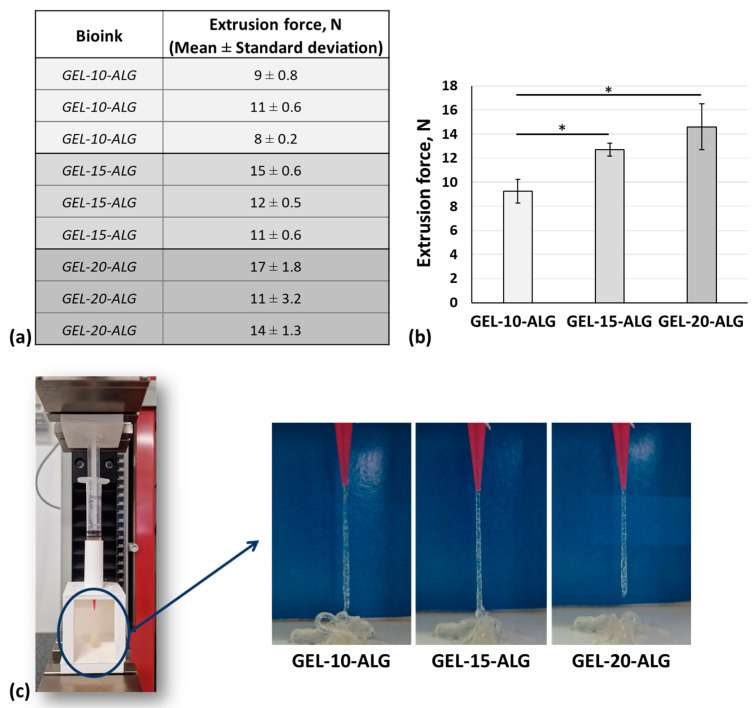
(**a**) Extrusion force reported as mean ± standard deviation calculated for each formulation tested in triplicate; (**b**) mean of extrusion force calculated for each production batch, * *p* < 0.05; (**c**) filament-like shape of extruded bioinks during the homogeneity assessment.

A dedicated setup was implemented to measure the extrusion force of each proposed formulation. In particular, the test was carried out on three different production batches of each formulation to evaluate the homogeneity of each production batch and the batch-to-batch reproducibility. In Figure 3a, the mean value of extrusion force obtained for each formulation is reported. If a constant displacement is applied to a homogenous solution during the extrusion process, the solution homogeneity will result in a constant extrusion force. Therefore, the extrusion force is constant for the formulation prepared with 10 and 15% of gelatin, while the formulations with 20% are characterized by a high standard deviation; therefore, the extrusion force cannot be considered constant. Regarding batch-to-batch reproducibility, the graph of the mean value of the extrusion force calculated on three different batches for each formulation is shown in Figure 3b. The mean value of the extrusion force increases according to the gelatin concentration, and the formulations with 10 and 15% of gelatin are reproducible.

Moreover, all the formulations can be extruded with a filament-like shape, as shown in Figure 3c.

### 2.3. Printability

The printability assessment was performed at room temperature, and the extrusion pressure and printing speed, set following the preliminary experiment, are reported in Table 1.

**Figure 4:**
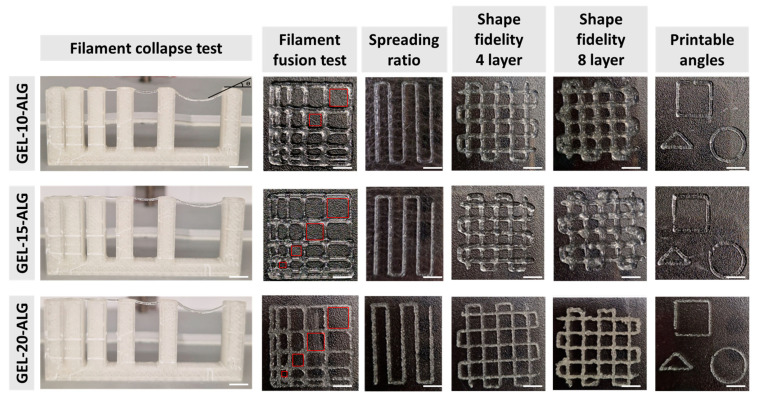
Printability assessment of proposed bioink formulation performed through filament collapse test, filament fusion test, spreading ratio, shape fidelity, and printable angle test.

The filament collapse test was performed using a custom platform with six supports at gap distances of 1, 2, 4, 8, and 16 mm. Figure 4 shows the images acquired immediately after a filament printing formulation on the platform. No filament deflection was observed between supports at distances less than 4 mm, while a filament deflection was observable between supports placed at the distances of 8 and 16 mm for all formulations. In particular, the deflection angle between the supports at a distance of 8 mm is equal to 8° for GEL-10-ALG, 7° for GEL-15-ALG, and 10° for GEL-20-ALG. Filament deflection angles of 19° for GEL-10-ALG, 18° for GEL-15-ALG, and 27° for GEL-20-ALG were measured between supports placed at 16 mm. The deflection angle increases as the distance between the supports increases and according to the concentration of gelatin present in the bioink formulation. However, a complete collapse of the filament was not observed for all tested formulations.

A dedicated model characterized by macropores of square or rectangular geometries with variable dimensions was printed to evaluate the minimum printable space between the filaments and, therefore, the bioink resolution. As shown in Figure 4, a fusion of the filaments placed at 1 mm on the x and y axis, resulting in the closure of the macropores, was observed. The GEL-10-ALG bioink is characterized by a partial fusion, even of the filaments placed at 2 and 3 mm of distance, while GEL-15-ALG and GEL-20-ALG do not present further fusions between the filaments.

Representative images of the structures printed using the three proposed gelatin-based bioinks, employed for the spreading ratio (SR) and shape fidelity (*Pr*) calculation, are reported in Figure 4. The ideal value for both parameters is 1, and the GEL-15-ALG bioink has a spreading ratio value that is closest to the ideal value (Table 1); moreover, the edges of the filament are smooth and with a uniform finish. The shape fidelity values, evaluated on four overlapping layers, are similar for all formulations tested and are better than the shape fidelity values measured in the structures printed with eight overlapping layers.

The versatility of the bioinks in terms of different printable shapes was evaluated by printing a square, a triangle, and a circle (Figure 4). The proposed bioink formulations allowed for the printing of different shapes with replications of the 45° and 90° angles for the triangle and the square, respectively, confirming the versatility of the bioinks.

### 2.4. Morphology

The morphological structure of the samples printed using the different formulations (10, 15, and 20% of gelatin) and crosslinked with 100, 150, and 200 mM of CaCl_2_ solutions were evaluated.

**Figure 5:**
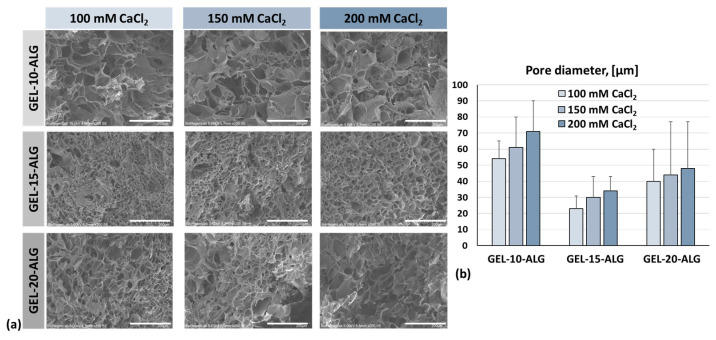
(**a**) SEM images of printed and crosslinked constructs, scale bar 200 µm; (**b**) pores diameter.

Figure 5a shows the SEM images of the structure for all proposed combinations of bioinks and crosslinking solution, while the diameters of the measured pores are reported in Figure 5b.

There is no significant increase (*p* ≤ 0.05) in the pore diameter in GEL-10-ALG compared to GEL-15-ALG and GEL-20-ALG. The GEL-15-ALG bioink has pores of smaller diameters than the other two proposed formulations, but presents greater homogeneity and a denser and more porous internal structure. The GEL-10-ALG and GEL-20-ALG bioinks have variable pore diameters and, therefore, a less homogeneous internal structure. Furthermore, for each formulation, there is no significant increase (*p* ≤ 0.05) in the pore diameter when the concentration of CaCl_2_ used for crosslinking increases.

### 2.5. Degradation and Swelling

The in vitro degradation rate was evaluated during 35 days of incubation in complete culture medium at 37 °C and 5% CO_2_ to simulate the in vitro standard culture condition (Supporting Information, Figure S1).

The samples were bioprinted using the three proposed gelatin-based formulations, and each one was crosslinked using 100, 150, or 200 mM CaCl_2_ as the crosslinking solution. All tested samples are characterized by an initial increment of weight with a subsequent weight reduction that starts at different time points: 10 days for GEL-10-ALG and 17 for GEL-15-ALG and GEL-20-ALG. The gelatin and crosslinking agent concentrations do not significantly influence the degradation rate.

Anyway, a complete degradation of samples occurs at day 35 for GEL-20-ALG, while GEL-10-ALG and GEL-15-ALG show 20% and 60% of the mass is retained.

The swelling index, defined as the ratio between the hydrated and dried weight of the sample, was calculated for the proposed bioink formulations at 1, 4, 6, 24, and 28 h of incubation in deionized H_2_O (Supporting Information, Figure S2). For each gelatin-based formulation, there are no significative differences among the three crosslinking solutions tested; however, the samples bioprinted with 15% and 20% of gelatin are characterized by lower swelling indexes than the GEL-10-ALG, resulting in lower water uptake.

### 2.6. Three-Dimensional Bioprinting of a Skin Model

Human primary fibroblasts NHDFs and human keratinocytes HaCaTs were added to the GEL-15-ALG bioinks at a final concentration of 3 × 10⁶ cells/mL and 4 × 10⁶ cells/mL, respectively. The constructs, composed of two overlapped layers, were crosslinked immediately after bioprinting with 150 mM CaCl_2_ for 10 min and were maintained in culture using high-glucose DMEM medium supplemented with 10% FBS, 1% glutamine, and 1% penicillin–streptomycin at 37 °C for 14 days.

**Figure 6:**
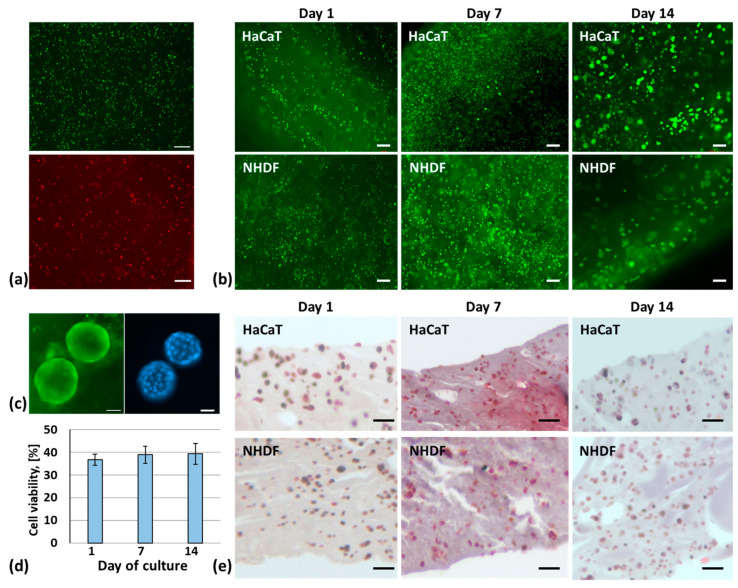
(**a**) Live/dead staining immediately after bioprinting, scale bar 200 µm; (**b**) live/dead staining at 1, 7, and 14 days of in vitro culture, scale bar 200 µm; (**c**) HaCaT clusters at day 14 of culture, scale bar 100 µm; (**d**) cell viability by resazurin assay, and (**e**) histological analysis, scale bar 200 µm.

The viability and proliferation of cells within the printed constructs were qualitatively assessed by a live/dead assay at days 1, 7, and 14 of culture. Figure 6a shows the live/dead staining of cells immediately after printing; the number of live cells (green) is greater than the number of dead cells (red), while Figure 6b shows the live/dead staining of NHDF fibroblasts and of HaCaT keratinocytes on days 1, 7 and 14. After 7 days of in vitro culture, the number of fibroblasts and keratinocytes in the constructs increased. At day 14, the fibroblasts took on an elongated shape, continuing to proliferate, while the keratinocytes formed clusters (Figure 6c).

The resazurin assay was performed (Figure 6d) to quantitatively evaluate cell viability. The increase in its reduced form, resorufin, is proportional to the number of viable cells within the construct. A cell viability of 36.8, 38.9, and 39.3% was measured, respectively, on days 1, 7, and 14 of culture, demonstrating a trend in proliferation, although there was no significant difference among the various time points.

Histological analysis was performed to evaluate the distribution of cells within the printed constructs. The Masson’s trichrome staining of the histological sections of the constructs fixed on days 1, 7, and 14 of culture is reported in Figure 6e. The staining allows for the clear identification of the cells, which correspond to the rounded structures present in the sections.

## 3. Discussion

In vitro skin models are receiving great attention from researchers and industries because they represent a potential alternative tool to traditional animal-based models to investigate basic skin biology as well as to study the effect of novel products [27]. Reconstructed human epidermises represent the majority of in vitro skin models; however, they lack a dermal layer and complete skin structure [28,29]. In this context, 3D bioprinting represents the most attractive biofabrication technology due to the possibility of spatial controls of cells and/or bioactive molecules laden bioink deposition using multiple printheads to produce skin models [28].

This study aimed to develop a gelatin-based bioink for the 3D bioprinting of a simplified skin model useful for the evaluation of the bioactivity of pro-reparative molecules and drugs. Three different bioink formulations containing 10, 15, or 20% of gelatin (GEL-10-ALG, GEL-15-ALG, and GEL-20-ALG, respectively) and 6% alginate semi-crosslinked with 100 mM CaCl_2_, added in a volumetric ratio of 25:9 to increase the viscosity of the formulations, were developed and characterized. Moreover, the effect of crosslinking concentrations (100, 150, or 200 mM of CaCl_2_) after bioprinting was investigated in terms of morphology, degradation, and swelling properties.

The post-printing polymerization is due to the interaction between the alginate molecules in the bioink and Ca^2+^ ions in calcium chloride solution. Therefore, the crosslinked formulation can be defined as a semi-interpenetrating polymer network (semi-IPN) composed of gelatin molecules dispersed only into the network formed by the crosslinked alginate chains without forming another network interpenetrated with the alginate one [30,31]. In this way, the properties of individual components are combined, obtaining a synergistic effect. A sodium alginate/gelatin blend as a bioink for extrusion bioprinting has been considered in many studies [13,23,32]; however, the study of the properties of bioinks modifying both the concentration of gelatin and CaCl_2_, used as a crosslinker, represents the novelty of this study compared to the state of the art.

Since standard sterilization methods adversely affect material polymer properties and cell viability, a bioink needs to be sterile before cell addition and skin model biofabrication. Despite the importance of bioink sterility, few studies have focused on this aspect [33,34]. In this study, the bioink formulations were prepared under sterile conditions using sterile precursor solutions. In particular, the alginate solution was sterilized by filtration [35] as well as the calcium chloride solution, while the sterile gelatin solution was obtained by autoclaving. Gelatin solution autoclaving causes a dramatic reduction in both yield stress and viscosity with respect to the untreated solution [35]; however, likely due to the selected gelatin concentration and alginate addition, a viscous formulation suitable for bioprinting was obtained. The sterility of proposed bioink formulations was verified by incubation in microorganism broth according to the literature [35,36].

All the proposed characterizations have been performed only on the gelatin–alginate-based formulations without considering the controls: the formulations produced using only alginate or gelatin. This is related to the data available on semi-crosslinked alginate-based formulations investigated in our previously published work [10] and to the non-printability of the gelatin solution at the highest considered concentration of 20% at 37 °C due to the low viscosity.

The rheological properties of gelatin-based formulations were investigated through the viscosity, storage modulus (G′), and loss modulus (G″) analyses at 25 °C and 37 °C. The proposed formulations showed a reduction in viscosity as the shear rate increased, confirming a shear-thinning behavior. The G′ is higher than the G″ in all three proposed formulations; therefore, the bioinks showed a gel-like behavior before crosslinking [37]. The gap between G′ and G″ is higher at 25 °C than 37 °C, probably due to the thermal gelation of gelatin. As expected, the values of viscosity, G′, and G″ are the lowest in the samples characterized at 37 °C rather than at 25 °C. All the formulations tested are characterized by a storage modulus G′ at 37 °C, which fits with the suggested range of 10^2^–10^3^ Pa for printable hydrogel [38]. In addition, as reported in the literature [26], the formulation with the highest gelatin amount is characterized by the highest values of viscosity, G′, and G″.

The semi-crosslinking of alginate was obtained by slowly pouring CaCl_2_ into the gelatin–alginate solution under stirring according to Falcone et al. [39]. The formulation homogeneity and the batch-to-batch reproducibility were assessed by measuring the extrusion force magnitude for each formulation using a custom syringe setup. Adding 100 mM CaCl_2,_ the resulting GEL-10-ALG and GEL-15-ALG were homogeneous and reproducible solutions, while GEL-20-ALG was inhomogeneous and, therefore, not reproducible. Despite this, all three formulations can be extruded with filament-like shapes, but the GEL-20-ALG can only be extruded in a discontinuous way. However, the mean value of extrusion force increases according to the gelatin concentration, confirming the results of rheological characterization.

Printability is a fundamental aspect of a bioink to obtain a 3D bioprinted structure that is able to provide a suitable environment for tissue regeneration [40]. Five different tests were performed to characterize the printability of proposed formulations. The printability is strictly correlated to the gelatin amount in the formulation. As expected by rheological characterization, adding a higher concentration of gelatin results in a solution with higher printability in terms of the lowest deflection angles, highest resolution and spreading ratio, and shape fidelity nearest to the ideal value of 1. However, considering that the GEL-20-ALG is not homogenous, the extruded filament is not smooth; therefore, the GEL-15-ALG represents the formulation with the best printability. This trend is also reported in the literature by Peng et al. [32]. In their study, the authors proposed different formulations of alginate bioink with increasing concentrations of gelatin up to 4%, and, among them, the formulation with 2% of gelatin results in better printability.

The pore size, architecture, and structure stability over time play an important role in tissue regeneration because they are essential to provide adequate mechanical support, optimized diffusivity of O_2_ and nutrients, and waste permeability [41]. Therefore, the effect of different concentrations of CaCl_2_ employed as crosslinking solutions after the bioprinting process was investigated through the evaluation of construct morphology, degradation rate, and swelling.

All the crosslinked constructs investigated in this study showed an internal structure with interconnected pores of irregular shape and heterogenic diameter. No significative differences have been observed in pore diameter among the tested samples; however, a trend between the pore diameter and calcium chloride amount in the crosslinking solution can be observed. In particular, by increasing the calcium chloride concentration, an increment in pore diameter was measured. This effect is probably related to the Ca^2+^ diffusion rate/bioink crosslinking front migration rate increment due to the Ca^2+^ crosslinker concentration increment, which could cause a reduction in Ca^2+^ ion diffusion within the 3D bioprinted structure [42]. The crosslinking with increased concentrations of CaCl_2_ did not cause the polymer chains to be more tightly packed and, consequently, did not cause a pore size reduction, similarly to Skopinska-Wisniewska et al., who observed an increment in the pore size [43].

Regarding the gelatin concentration effect, in our study, the increment in gelatin concentration from 10 to 15% causes a reduction in pore size, as previously observed by Serafin et al. [44]. At 20% of gelatin, the reduction in pore size with respect to 15% was probably caused by a reduction in the Ca^2+^ ion diffusion coefficient for temperatures below the gelatin gelling point [45], as observed also with the increment in viscosity in rheological characterization at 25 compared 37 °C. In addition, the different pore sizes could be related to a different semi-IPN structure formation due to the increment in gelatin concentration. However, the gelatin addition causes a variation in the internal structure with the presence of more spherical pores, with respect to alginate alone [10].

The degradation percentages of three tested gelatin-based bioinks were determined considering the weight loss of the analyzed samples. The formulation containing 20% of gelatin is characterized by a complete degradation in 35 days of in vitro culture, while the samples bioprinted with GEL-10-ALG and GEL-15-ALG are characterized by a mass loss of about 80 and 40%, respectively. The calculated degradation rates are probably related to the measured pore diameter, which is smaller in GEL-15-ALG samples. However, the in vitro culture is usually performed in a period of 14 or 21 days [3,46]. Thus, the degradation rate is considered acceptable.

All proposed formulations have a high water uptake (100% or more), as confirmed by swelling. The calcium chloride concentration did not have a significant effect as observed for the degradation rate; however, the GEL-10-ALG, which is characterized by the highest pore diameter, is the formulation with the highest swelling index and, therefore, water uptake according to the literature [47].

Considering the superior printability properties, the lowest in vitro biodegradation, and swelling index, GEL-15-ALG crosslinked with 150 mM CaCl_2_ is the bioink formulation selected for the bioprinting of the simplified skin model.

The skin model is composed of two overlapping layers, one loaded with human primary fibroblast to mimic the dermal layer, and the other one with human keratinocytes to mimic the epidermal layer. Here, 3 × 10^6^ NHDF/mL were added to GEL-15-ALG bioink for dermis printing, while 4 × 10^6^ HaCaT/mL were added to another aliquot of GEL-15-ALG bioink for epidermis printing. The skin models were crosslinked with 150 mM CaCl_2_ for 10 min and grown for 14 days in culture medium, and cell viability was assessed at specific time points. The protocol used for embedding cells into the bioink, as well as the selected bioprinting parameters (extrusion pressure of 13 kPa, print head speed of 4 mm/s, and a 22 G nozzle), does not affect cell viability, as demonstrated by the images captured on samples immediately after printing. The live/dead assay showed that the number of green viable cells is qualitatively higher with respect to red dead cells for both fibroblasts and keratinocytes. Moreover, the slow rate of degradation observed in cell-free samples was also qualitatively observed in cell-laden samples for up to 14 days; in fact, at the end of the incubation period, samples are yet to be easily handled, despite a faster degradation rate being reported in the literature for cell-laden rather than cell-free bioinks [48,49].

The live/dead assay showed a proliferation of fibroblasts and keratinocytes on days 7 and 14 of culture, compared to previous time points of analysis. These findings were quantitatively confirmed by the resazurin reduction, demonstrating that the pore size of the bioink is suitable for cell proliferation. Furthermore, the fibroblasts after 14 days of culture showed the typical morphology with an elongated shape, while the keratinocytes formed numerous clusters with a wide range of diameters. Cell addition to bioink is a critical factor of the bioprinting process because the addition of cells can alter the physical properties of the bioink, affecting the printability and shape fidelity of the final construct [50,51]. In this study, cell addition does not change the bioink printability; the printed constructs have a structure composed of two distinct layers, one containing fibroblasts and the other keratinocytes, as observed in the thickness of the construct by histological sections. Masson’s trichrome staining was successfully used to stain cell nuclei without staining the bioink, while the standard Hematoxylin and Eosin stains the bioink, precluding the discrimination of cells.

Regarding the epidermal layer, a non-confluent layer of keratinocytes was observed in the present construct, probably due to the used cell density that plays an important role in cell–cell signaling and the regulation of cell differentiation. Therefore, increasing the cell density for each cell type could allow for obtaining better results in terms of cell proliferation, as previously demonstrated in our study on a fibrinogen-based bioink development [11] and by other authors on a gellan gum-based and on alginate–gelatin–graphene oxide [52,53] bioinks. In addition, by combining the cell density with a longer culture period of at least 21 days, better results could be obtained and a deeply biological characterization could be performed to investigate the tissue maturation/ECM deposition [54,55].

## 4. Materials and Methods

### 4.1. Bioink Preparation

Gelatin-based bioinks were prepared by dissolving gelatin powder extracted from porcine skin (50–100 kDa, Merck KGaA, Darmstadt, Germany) at 10, 15, and 20% (*w*/*v*) in deionized water. The solutions were autoclaved to obtain gelatin solubilization and sterilization. Sodium alginate (Merck, 120–190 kDa) was dissolved at 2% in distilled water, sterilized by filtration (0.22 μm), and subsequently freeze-dried [10,30]. Then, the obtained sterile alginate powder was added to the gelatin solutions with a concentration equal to 6% (*w*/*v*) and stirred for 15 min at 50 °C. A sterile 100 mM calcium chloride (CaCl_2_, Merck) solution was added to the alginate/gelatin at a volumetric ratio of 25:9 and stirred for 10 min at 50 °C to obtain semi-crosslinked solutions. The solutions were centrifuged at 3000 rpm for 15 min to remove air bubbles and stored at 4 °C until use. The final concentration of each bioink formulation is shown in Table 2.

Crosslinking solutions were prepared dissolving CaCl_2_ powder in deionized water at concentrations of 100, 150, and 200 mM. The crosslinking was performed by sample submersion in crosslinking solutions for 10 min.

The following characterizations were performed on three different production batches for each gelatin-based bioink formulation.

### 4.2. Sterility Evaluation

The sterility of the gelatin-based bioinks was assessed using a broth for the cultivation of microorganisms. Briefly, sterilized gelatin-based samples were immersed in Mueller–Hinton broth (Oxoid Ltd., Basingstoke, UK) and incubated at 37 °C overnight. Sterile broth was used as the negative control, while intentionally contaminated samples served as the positive control. Any clouding of the broth indicated contamination and inefficient sterilization process, while a clear, uncontaminated broth indicated an efficient sterilization process.

### 4.3. Rheological Properties and Homogeneity

Alginate and gelatin solutions with the addition of CaCl_2_ were placed in different vials, and the semi-crosslinking effect was evaluated using the vial-inverting method. Samples without CaCl_2_ were used as reference.

The viscosity, storage (G′), and loss (G″) modulus were calculated for rheological property assessments. Rotational tests (shear rate sweep) and oscillatory tests (amplitude sweep and frequency sweep) were performed at 25 °C and 37 °C using a modular compact rheometer (MCR 302, Anton Paar, Turin, Italy) equipped with a plate–plate geometry (Ø = 5 cm) and a protective hood, as previously described [10].

The bioinks’ homogeneity was assessed using the setup previously described [10]. Briefly, each batch of gelatin-based bioink was loaded into a 3 mL syringe equipped with a 22 G nozzle and vertically mounted into the setup composed of a custom syringe holder and a Zwick-Roell Z1.0 testing machine equipped with a 100 N load cell (Zwick GmbH & Co., Ulm, Germany). A constant displacement was applied to the syringe plunger while the extrusion force was measured.

### 4.4. Printability

**Figure 7:**
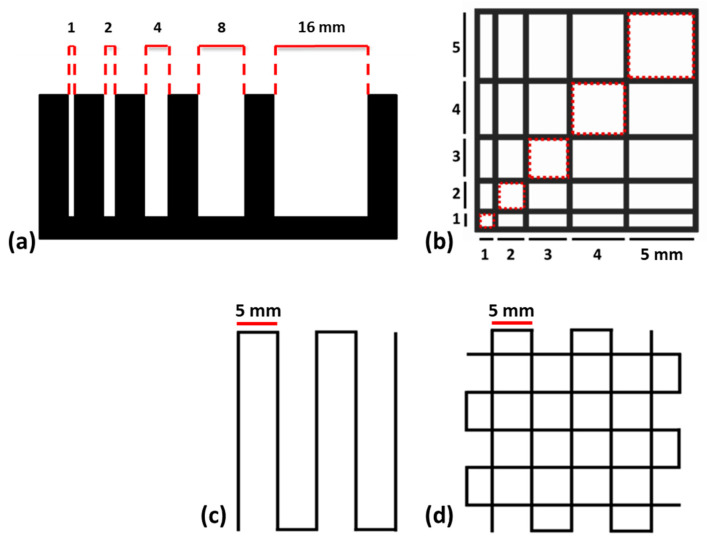
(**a**) Custom platform for filament collapse test; (**b**) pattern for filament fusion test; (**c**) pattern for spreading ratio assessment; and (**d**) pattern for shape fidelity assessment.

Five different tests were performed at room temperature to characterize the gelatin-based bioinks printability [10,11] (each test was carried on in triplicate): (i) filament collapse test performed by printing a bioink fiber on a custom platform (Figure 7a) composed of pillars with different gap distances (1, 2, 4, 8, and 16 mm); (ii) inter-filament line spacing evaluated on a pattern characterized by a linear increase in line spacing in both X and Y directions (Figure 7b); and (iii) shape fidelity (*Pr*), calculated according to the following equation using a dedicated pattern (Figure 7c) composed of 4 or 8 overlapped layers:

$$Pr=\frac{L^{2}}{16A}$$

where *L* is the interconnected pore perimeter and *A* is the area; (iv) spreading ratio calculated as the ratio between the extruded filament width measured on the bioprinted pattern (Figure 7d) and the nozzle diameter; and (v) printable shape.

The printability tests were carried out using the 3D BioX (Cellink, Goteborg, Sweden) as a bioprinter equipped with an extrusion printhead-loaded 3 mL cartridge with a 22 G nozzle.

### 4.5. Morphology

The microporosity was evaluated for each bioink formulation using different crosslinking solutions, namely 100, 150, or 200 mM CaCl_2_, after bioprinting. Samples of 10 mm in diameter and 1 mm in thickness were bioprinted, crosslinked, lyophilized for 24 h (a 2-step slow freezing process was employed at −20 °C and then at −80 °C), and sputter coated before image acquisition observed at scanning electron microscopy (FlexSEM 1000, Hitachi, Tokyo, Japan). Images were captured at an accelerating voltage in the range of 5–15 kV on two different samples. Three randomly captured images of each sample were used to evaluate the pore sizes (30 measures in each image) using open-source ImageJ 1.52 software (National Institutes of Health, Bethesda, MD, USA).

### 4.6. Degradation and Swelling

Samples (six replicates for each characterization test) 8 mm in diameter and 2 mm in thickness were bioprinted using each gelatin-based formulation and submerged in 100, 150, or 200 mM CaCl_2_ as crosslinking solution.

The degree of degradation was calculated according to the following formula:

$$\mathrm{degradation}\;\mathrm{rate}\;(\%)=\frac{\left(W0-Wt\right)}{W0}\times 100$$

where *W*0 is the weight of samples immediately after crosslinking and *Wt* is the sample weight after degradation time *t*. Samples were incubated in high-glucose DMEM cell culture medium supplemented with 10% fetal bovine serum (FBS), 1% glutamine, and 1% penicillin–streptomycin (defined as complete culture medium) at 37°C and 5% CO_2_ to simulate the cell culture and were weighted every two days for the 30 days of incubation.

The swelling index was evaluated on lyophilized samples according to the following formula:

$$\mathrm{SI}=\frac{Wh}{Wd}$$

where *Wh* is the weight of hydrated samples, while *Wd* is the weight of lyophilized samples. The samples were incubated in deionized H_2_O for 48 h and weighed at 1, 4, 6, 24, and 28 h of incubation.

### 4.7. Three-Dimensional Bioprinting of a Skin Model

Normal human dermal fibroblasts (NHDFs, PromoCell, Heidelberg, Germany) and human keratinocytes (HaCaTs, Istituto Zooprofilattico Sperimentale della Lombardia e dell’Emilia Romagna “Bruno Ubertini”, Brescia, Italy) were cultured in high-glucose DMEM at 37 °C, 5% CO_2_. The medium was changed every 3 days, and the cells were split at 70–80% confluence.

Here, 3 × 10^6^ NHDF/mL and 4 × 10^6^ HaCaT/mL were added to two different bioink aliquots containing 15% of gelatin. Briefly, cells were harvested using 1% trypsin-EDTA (Merck), pelleted, and resuspended in 100 µL of medium before addition to the bioink using two syringes connected using a female/female luer lock adapter. The cell-laden bioinks were charged into different cartridges equipped with 22 G nozzles. The samples, biofabricated with an extrusion pressure and temperature of 13 kPa and 37 °C, respectively, and a printing rate of 4 mm/s, have a cylindrical shape (diameter of 8 mm and thickness of 2 mm) and are composed of two layers of keratinocytes and three layers of fibroblasts. After printing, the samples were crosslinked by submersion in 600 μL of CaCl_2_ solution (150 mM) for 10 min and then incubated in 600 μL of high-glucose DMEM for up to 14 days.

### 4.8. Cell Viability and Proliferation

The live/dead assay (Merck) was performed to qualitatively evaluate cell viability, morphology, and distribution within the 3D bioprinted skin model. The assay involves the use of three fluorescent dyes: Calcein-AM, which stains the cytoplasm of live cells green, propidium iodide, which stains the nucleus of dead cells red, and Hoechst 33342, which stains the nuclei of living and dead cells blue. Immediately after printing and subsequently on days 1, 7, and 14 of culture, the samples were incubated in 500 µL of the staining solution for 45 min and observed with a fluorescence stereomicroscope equipped with z-stack (Zeiss Axio Zoom.V16, Carl Zeiss, Oberkochen, Germany), Zeiss digital camera (Axiocam 105 colors) and image processing software (Zen Blue, Carl Zeiss).

The resazurin assay, also known as the Alamar Blue assay, was used to quantitatively evaluate cell viability and cell proliferation within the constructs at different time points. Resazurin is a fluorescent dye that is metabolized by live cells into resorufin (reduced form). Since the resazurin assay keeps the cells viable, it is possible to perform the assay onto the same constructs at subsequent time points. Briefly, on days 1, 7, and 14 of culture, 50 µL of resazurin 1 mg/mL (Merck) and 500 µL of complete DMEM were added to each well. After 4 h of incubation, the absorbance was measured at 550 and 620 nm using a microplate reader (SpectraFluor Plus; TECAN Austria GmbH, Grödig, Austria).

### 4.9. Histological Analysis

Additionally, 3D bioprinted constructs were fixed in 4% paraformaldehyde solution in 50 mM CaCl_2_ for 20 h on days 1, 7, and 14 of culture. Samples were washed twice in PBS, dehydrated in ethanol series, diaphanized in xylene, and paraffin-embedded. Sections of 7 µm were cut using a microtome (HM350S, Microm), placed on Poly-L-Lysine-coated slides with balsam, and stained with Masson’s trichrome (Bio-Optica, Milan, Italy).

### 4.10. Statistical Analysis

Data are presented as the mean ± standard deviation of at least three independent experiments. Statistical analysis was carried out using StatViewTM 5.0 (SAS Institute, Cary, NC, USA). Statistical significance was determined by one-way ANOVA for rheology, extrusion force, printability, and cell viability data analysis and by two-way ANOVA for morphological, degradation, and swelling data analyses. Values were considered significant at *p* < 0.05.

## 5. Conclusions

In conclusion, in this study, a gelatin-based bioink that can be crosslinked using a 150 mM CaCl_2_ solution is proposed for the 3D bioprinting of an in vitro simplified skin model. The obtained results demonstrated the gelatin-based bioink printability and its ability to support fibroblast and keratinocyte viability and proliferation. The limitations of this study concern the lack of mechanical characterization that could provide information about ECM synthesis, and the post-printing period should be extended by up to 21 days to allow for a more complete model maturation. Despite the fact that more investigations should be performed from a biological perspective, by also adding more cell types, such as endothelial cells, to introduce vascularization, the obtained results could pave the way for a novel in vitro skin model that is useful to investigate the effects of drugs and bioactive molecules.

## Figures and Tables

**Table 1:** Printing parameters and calculated values for spreading ratio (SR) and shape fidelity (*Pr*).

Bioink	Extrusion Pressure, [KPa]	Printing Speed, [mm/s]	SR	*Pr* 4 Layers	*Pr* 8 Layers
GEL-10-ALG	10	4	2 ± 0.3	0.97 ± 0.2	0.94 ± 0.1
GEL-15-ALG	17	6	1.2 ± 0.2	0.95 ± 0.3	0.92 ± 0.2
GEL-20-ALG	32	9	1.5 ± 0.1	0.99 ± 0.1	1.12 ± 0.1

**Table 2:** Gelatin-based bioink compositions.

Bioink	Gelatin, [%]	Alginate, [%]	CaCl_2_, [mM]
GEL-10-ALG	7.4	4.4	26
GEL-15-ALG	11	4.4	26
GEL-20-ALG	14.8	4.4	26

## Data Availability

The original contributions presented in this study are included in the article/Supplementary Material. Further inquiries can be directed to the corresponding author.

## Supplementary Materials

The following supporting information can be downloaded at https://www.mdpi.com/article/10.3390/molecules30030649/s1, Figure S1: Degradation rate of 3D bioprinted samples using GEL-10-ALG, GEL-15-ALG, and GEL-20-ALG crosslinked with 100, 150, or 200 mM of CaCl_2_ solution; Figure S2: Swelling Index of 3D bioprinted samples using GEL-10-ALG, GEL-15-ALG, and GEL-20-ALG crosslinked with 100, 150, or 200 mM of CaCl_2_ solution.
