# Supplementary material for: Optimization of Gelatin and Crosslinker Concentrations in a Gelatin/Alginate-Based Bioink with Potential Applications in a Simplified Skin Model
Source: Molecules. 2025 Feb 1;30(3):649. doi: 10.3390/molecules30030649 (PMC11820930; doi:10.3390/molecules30030649)
Supplement: Supplementary file 1 [file molecules-30-00649-s001.zip › molecules-3385125-supplementary.pdf]

*Supplementary Materials*

# Optimization of Gelatin and Crosslinker Concentrations in a Gelatin/Alginate-Based Bioink with Potential Applications in a Simplified Skin Model

**Aida Cavallo [1,*], Giorgia Radaelli [1], Tamer Al Kayal [1], Angelica Mero [2], Andrea Mezzetta [2], Lorenzo Guazzelli [2], Giorgio Soldani [1] and Paola Losi [1]**

[1] Institute of Clinical Physiology, National Research Council, 54100 Massa, Italy; giorgia.radaelli99.gr@gmail.com (G.R.); tamer.alkayal@cnr.it (T.A.K.); giorgio.soldani@cnr.it (G.S.); paola.losi@cnr.it (P.L.)

[2] Department of Pharmacy, University of Pisa, 56126 Pisa, Italy; angelica.mero@farm.unipi.it (A.M.); andrea.mezzetta@unipi.it (A.M.); lorenzo.guazzelli@unipi.it (L.G.)

* Correspondence: aidacavallo@cnr.it; Tel.: +39-0585-483745

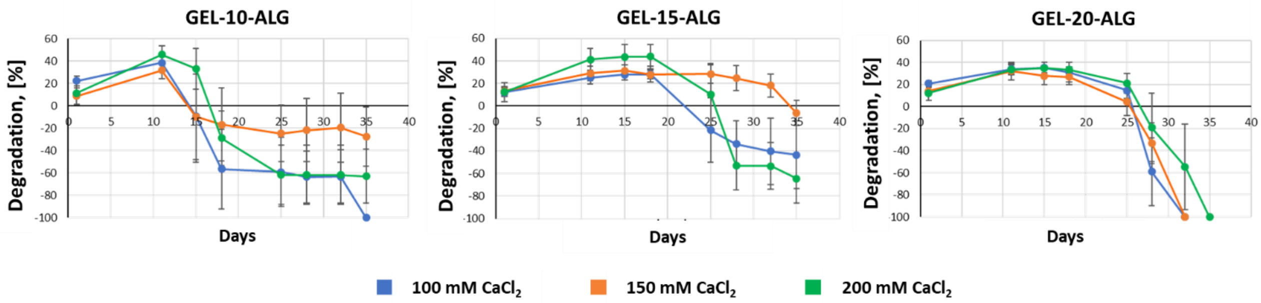


**Figure S1.** Degradation rate of 3D bioprinted samples using GEL-10-ALG, GEL-15-ALG, and GEL-20-ALG crosslinked with 100, 150, or 200 mM of $CaCl_2$ solution.

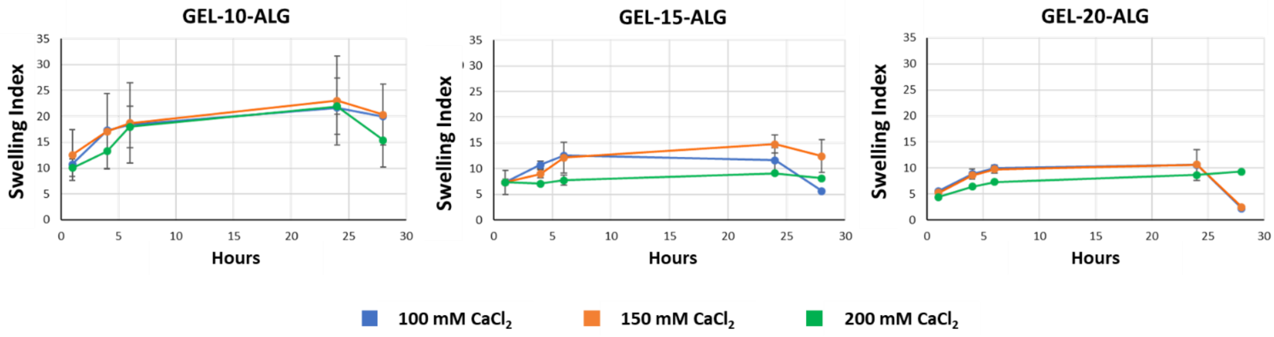


**Figure S2.** Swelling Index of 3D bioprinted samples using GEL-10-ALG, GEL-15-ALG, and GEL-20-ALG crosslinked with 100, 150, or 200 mM of $CaCl_2$ solution.
